# Cost-effectiveness of community-based strategies to strengthen the continuum of HIV care in rural South Africa: a health economic modelling analysis

**DOI:** 10.1016/S2352-3018(15)00016-8

**Published:** 2015-04

**Authors:** Jennifer A Smith, Monisha Sharma, Carol Levin, Jared M Baeten, Heidi van Rooyen, Connie Celum, Timothy B Hallett, Ruanne V Barnabas

**Affiliations:** a Department of Infectious Disease Epidemiology, Imperial College London, London, UK; b Department of Epidemiology, University of Washington, Seattle, WA, USA; c Department of Global Health, University of Washington, Seattle, WA, USA; d Department of Medicine, University of Washington, Seattle, WA, USA; e HIV/AIDS, STIs and TB, Human Sciences Research Council, Sweetwaters, KwaZulu-Natal, South Africa; f Vaccine and Infectious Diseases Division, Fred Hutchinson Cancer Research Center, Seattle, WA, USA

## Abstract

**Background:**

Home HIV counselling and testing (HTC) achieves high coverage of testing and linkage to care compared with existing facility-based approaches, particularly among asymptomatic individuals. In a modelling analysis we aimed to assess the effect on population-level health and cost-effectiveness of a community-based package of home HTC in KwaZulu-Natal, South Africa.

**Methods:**

We parameterised an individual-based model with data from home HTC and linkage field studies that achieved high coverage (91%) and linkage to antiretroviral therapy (80%) in rural KwaZulu-Natal, South Africa. Costs were derived from a linked microcosting study. The model simulated 10 000 individuals over 10 years and incremental cost-effectiveness ratios were calculated for the intervention relative to the existing status quo of facility-based testing, with costs discounted at 3% annually.

**Findings:**

The model predicted implementation of home HTC in addition to current practice to decrease HIV-associated morbidity by 10–22% and HIV infections by 9–48% with increasing CD4 cell count thresholds for antiretroviral therapy initiation. Incremental programme costs were US$2·7 million to $4·4 million higher in the intervention scenarios than at baseline, and costs increased with higher CD4 cell count thresholds for antiretroviral therapy initiation; antiretroviral therapy accounted for 48–87% of total costs. Incremental cost-effectiveness ratios per disability-adjusted life-year averted were $1340 at an antiretroviral therapy threshold of CD4 count lower than 200 cells per μL, $1090 at lower than 350 cells per μL, $1150 at lower than 500 cells per μL, and $1360 at universal access to antiretroviral therapy.

**Interpretation:**

Community-based HTC with enhanced linkage to care can result in increased HIV testing coverage and treatment uptake, decreasing the population burden of HIV-associated morbidity and mortality. The incremental cost-effectiveness ratios are less than 20% of South Africa's gross domestic product per person, and are therefore classed as very cost effective. Home HTC can be a viable means to achieve UNAIDS' ambitious new targets for HIV treatment coverage.

**Funding:**

National Institutes of Health, Bill & Melinda Gates Foundation, Wellcome Trust.

## Introduction

Expansion of the use of antiretroviral therapy is a major component of approaches to control the HIV epidemic, particularly vital in sub-Saharan Africa, where about 1·5 million new HIV infections occur each year.^1^ However, despite the high burden of disease, only a third of adults in sub-Saharan Africa have been tested for HIV in the past year, and less than a third of those testing positive receive antiretroviral therapy.^2–5^ Antiretroviral therapy substantially reduces HIV-associated morbidity and mortality and reduces the risk of transmission to susceptible partners by up to 96% through suppression of viral replication.^6–9^ For HIV treatment programmes, cost-efficient HIV counselling and testing (HTC) and approaches that link patients to care are urgently needed to prevent morbidity and mortality.

Traditional facility-based HTC, including voluntary counselling and testing and provider-initiated counselling and testing, has not attained high coverage of testing or antiretroviral therapy uptake in sub-Saharan Africa and will probably be insufficient to meet the ambitious 90-90-90 target set by UNAIDS, which aims by 2020 for 90% of HIV-infected individuals to be diagnosed, 90% of patients with a diagnosis to have initiated treatment, and 90% of those to be virally suppressed.^10^ Coverage of testing varies substantially by country. For example, findings from recent studies have shown that 17% of men and 17% of women in Nigeria, 36% and 47% in Kenya, 51% and 72% in Malawi, and 65% and 68% in South Africa had been tested in the previous 12 months and received their results.^11–14^ Furthermore, national coverage rates often mask large subnational variation. Barriers to HIV testing include economic costs for patients (transportation, waiting time, childcare), concerns about confidentiality, and low perceived risk of HIV infection.^15^ The policy of antiretroviral therapy eligibility at a CD4 count of 350 cells per μL or lower might have also encouraged providers and people living in the community to wait until they are clinically ill before seeking testing or treatment. Moreover, many clinics have poor linkage to treatment for individuals tested and found eligible.^16^

Community-based HTC (ie, HTC outside of facilities), which includes home-based, door-to-door, and mobile-van testing,^10^ reaches more first-time testers and HIV-positive individuals with high CD4 cell counts than does facility-based HTC. Community-based HTC also relies less heavily on existing infrastructure, allowing for easier scale-up.^10^ Community HTC with mobilisation and enhanced linkage to care has the potential to overcome barriers to HIV testing and linkage to care, achieving widespread coverage of testing and antiretroviral therapy.^2,17,18^ Strategies for community HTC and linkage have high acceptability and lead to greater uptake of testing, linkage to care, and viral-load suppression than does facility-based HTC.^10,19–21^ Large community-randomised controlled trials are underway to assess the effect of home HTC with enhanced linkage to care on uptake of antiretroviral therapy and HIV incidence.^21–24^ However, key questions remain about the affordability and cost-effectiveness of community HTC compared with current practice.

The costs of community-based HTC vary by country, testing method, and HIV prevalence. Published cost estimates for sub-Saharan Africa range from US$2·45 per person tested in Malawi (cost of testing supplies only) to between $81·72 and $126·48 in South Africa (cost of testing supplies, personnel, utilities, training, buildings, office equipment, and publicity for church-based HTC).^25,26^ Mobile HTC with point-of-care CD4 testing in South Africa ranged from $29·30 to $31·30 and facility-based testing ranged from $9·30 in South Africa to $29·56 in Kenya for testing at a fixed HTC site.^27,28^ Accurate cost data are crucial for estimating potential budgetary effects of implementing widespread HTC and linkage programmes.

Home HTC with point-of-care CD4 testing (with the PIMA analyser [Alere, Waltham, MA, USA]) and lay counsellor follow-up visits to encourage linkage to care can achieve high HTC coverage (91%), linkage to care (90%), and antiretroviral therapy initiation (80% of those eligible) in the high-HIV-prevalence setting of KwaZulu-Natal, South Africa.^19,29^ Early diagnosis and treatment reduces morbidity, mortality, and onwards transmission.^6–9^ We postulated that, despite the extra resources needed to implement these changes, a home HTC package including community mobilisation and sensitisation, point-of-care CD4 testing, screening for clinical indicators for antiretroviral therapy initiation, and follow-up visits by a community health worker (to support uptake of antiretroviral therapy and adherence) could avert additional morbidity and mortality compared with existing programmes. In this health economic modelling analysis, we aimed to estimate the population-level health effects of home HTC with facilitated linkage to care, and then use costing data from our previous studies of home HTC and care linkages to estimate the costs, cost-effectiveness, and feasibility of a home HTC intervention under simulated expansion of eligibility criteria for antiretroviral therapy.

## Methods

### Mathematical model

We parameterised an individual-based mathematical model with demographic, behavioural, and treatment uptake data from two studies of home HTC done in rural KwaZulu-Natal and calibrated to HIV incidence from a population survey (appendix).^18,19,30,31^ The full model specification is available in the appendix. Briefly, the model simulated a community of 1300 households with a total mean population size of 10 000 adults aged 18 years and older, capturing the characteristics (sex, age, and position in the household) of individuals enumerated in the home HTC project. Sexual behaviour, partnership formation and dissolution, and seasonal migration for work were distributed according to these characteristics. Children and teenagers enumerated in the pilot survey entered the model as they reached 18 years of age and all individuals in the model were subject to an age-specific and sex-specific background mortality rate (non-HIV-related) consistent with that in South Africa before the onset of the HIV epidemic.^32^

Sexual partnerships could be formed between any two adults of the opposite sex with a tendency for the male partner to be slightly older than the female partner (modal age difference 0–4 years in the study; unpublished data). Long-term partnerships had a mean duration of 7·15 years and could only be formed within the community. Short-term partnerships had a mean duration of 3 months and were preferentially formed with adults in the community; if none were available, short-term partners outside the community could be simulated. Adults outside the community were not explicitly modelled but had a probability of HIV infection based on the age and sex distribution of HIV prevalence reported in the home HTC studies. Individuals could have a maximum of two concurrent partners at any time, only one of which could be long-term.

HIV transmission was assumed to vary by sex, CD4 cell count (as a correlate of infection stage and viral load), antiretroviral therapy status of the HIV-infected partner, coital frequency (which varies according to partnership type), condom use, male circumcision, and co-infection with sexually transmitted infections of either partner. Condom use was distributed by partnership type and HIV status (appendix) and could change with long-term partnership formation or dissolution or a positive HIV test. After HIV infection, the model tracked fall in CD4 cell count for individuals through to death.

We assigned disability-adjusted life-years (DALYs) to each HIV-related health state, and summed them over all individuals for the duration of the model run (appendix).^33^ All scenarios started with the reported coverage of antiretroviral therapy of 32% among all HIV-positive individuals in the community at baseline. For both the intervention and non-intervention scenarios, we modelled four initiation thresholds for antiretroviral therapy: CD4 count 200 cells per μL or lower, 350 cells per μL or lower, 500 cells per μL or lower, and universal antiretroviral therapy (in which all HIV-positive people would be eligible for treatment).

### Procedures

**Figure 1:**
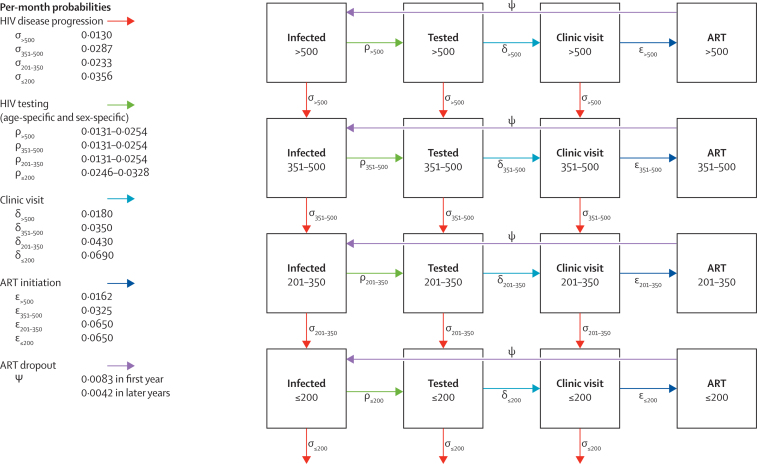
Flow diagram of HIV disease progression and the HIV care cascade among HIV-positive individuals HIV-positive individuals progress through HIV disease stages and on to HIV-related death at rates σ (top to bottom); subscripts indicate the CD4 cell count category to which the rate applies. Infected individuals (and uninfected, not shown here) are tested at rate ρ, attend clinics for CD4 staging and other clinical tests at rate δ, and initiate antiretroviral therapy at rate ɛ. Antiretroviral therapy dropout occurs at rate ψ irrespective of CD4 category at antiretroviral therapy initiation and individuals return to their previous CD4 count unlinked to care. Home HTC acts by instantaneously moving all eligible and untested individuals into the tested compartments, increasing rate δ for clinic visits, increasing rate ɛ for antiretroviral therapy initiation, and decreasing rate ψ for antiretroviral therapy dropout. Rate changes last for 1 year post-intervention, after which treatment-seeking behaviour reverts to the status quo. Note that HIV infection, HIV testing by uninfected individuals, natural mortality, and mortality on antiretroviral therapy are not shown here. HTC=HIV counselling and testing. ART=antiretroviral therapy.

In the status quo scenario (ie, without the intervention), the model simulated existing uptake of HIV testing and antiretroviral therapy to match the empirical data reported from the study at baseline.^19^ Individuals had a monthly probability of HTC that depended on their sex, infection status, and CD4 cell count if infected (appendix). For uninfected individuals, repeat HIV testing could occur a minimum of 1 year after a negative test, with the same monthly probability as initial testing. After a positive test, HIV-positive individuals had a monthly probability of linking to care that depended on their CD4 cell count (figure 1, appendix).

Once linked, we assumed that individuals were in regular pretreatment care and initiated antiretroviral therapy after a mean wait of 1·23 months after they became eligible. No difference in uptake rates for treatment were reported between individuals with CD4 counts of 200 cells per μL or lower and those with counts of 201–350 cells per μL in the field studies (data not shown), so we assumed that the mean wait for treatment remained constant when the threshold for treatment initiation changed to 350 cells per μL or lower. At higher eligibility criteria, we assumed longer wait times for individuals with CD4 counts of 351 cells per μL and above, on the assumption these individuals would be less likely to link to care than would those below the threshold;^29,30^ we conservatively assumed the monthly probability of antiretroviral therapy initiation for eligible individuals in pretreatment care (initially 6·5% per month) to halve for individuals with CD4 counts of 351–500 cells per μL and quarter for those with CD4 counts higher than 500 cells per μL (figure 1, appendix). In field studies we noted very few people initiating antiretroviral therapy with CD4 cell counts above the initiation criteria (data not shown), so in the model we assumed that only individuals with counts below that threshold could start treatment.

Home HTC was added to the status quo scenario at 91% coverage,^19^ repeated every 4 years (on the basis of findings from previous modelling analyses^34^), and resulted in an immediate HIV test and increased probability of linkage to care for HIV-positive individuals (monthly probability of 67% irrespective of CD4 count). At antiretroviral therapy eligibility criteria of 200 cells per μL or lower and 350 cells per μL or lower, we assumed the probability of treatment initiation for eligible individuals after home HTC to be 30% per month (fitted to data from the field studies). We used the same assumption as the status quo scenario when simulating higher criteria; the monthly probability of treatment initiation for eligible individuals in pretreatment care was assumed to halve for individuals with CD4 counts of 351–500 cells per μL and quarter for those with CD4 counts higher than 500 cells per μL (appendix). These changes lasted for 1 year after the intervention, after which testing and treatment-seeking behaviour returned to that in the status quo scenario. Individuals who had an HIV test in the previous 6 months or who were already receiving antiretroviral therapy were not retested by the intervention. Home HTC was modelled to reach 92% of women and 79% of men (on the basis of numbers of ineligible individuals from the field studies), taking into account migration for work. Condom use could increase after a positive HIV test (appendix).

We separated the intervention into constituent parts (increased testing, linkage to care, and antiretroviral therapy initiation) and estimated the contribution of each element to the overall effect.

We did 5000 model simulations for each scenario. We present summary results over 10 years as the median outcome bounded by the 5th and 95th percentiles (encompassing 90% of variability in model outputs).

### Cost analysis

We did a microcosting study in KwaZulu-Natal, South Africa, to derive home HTC costs during an ongoing community-based HTC trial (table 1).^35^ We adopted a provider perspective and collected costs from interviews with staff and local experts, budgets, expense reports, and travel logs. We did a mixture of ingredient-based and activity-based costing. We divided costs into mutually exclusive categories of personnel, transportation, equipment, supplies, buildings and overhead, start-up, recurring meetings, and data capture and use. We did time-and-motion studies to determine staff time spent on intervention activities (to estimate the mean number of visits that could feasibly be done each day), and to assess research time costs (time spent obtaining informed consent, reimbursements, etc). We annualised capital costs, software development, and start-up costs (eg, staff hiring, training, and community mobilisation) assuming a usable life of 5 years. Costs were discounted annually at 3%. We based background costs of HIV testing and linkage, health-care use, and antiretroviral therapy on estimates for South Africa (table 2).^33^

We defined three cost models to assess the importance of key assumptions. The first represented the actual costs of the research study, including staff and equipment used in the pilot study. The second was an operational costing model, omitting research-related costs, assuming task shifting from professional counsellors to lay staff (eg, community care workers), and increasing testing efficiency and using government salaries instead of research salaries for staff (table 1). The third was an operational costing model with reduced costs for antiretroviral therapy in South Africa (decreasing cost estimates for antiretroviral therapy from $565 per person-year for total related and non-related costs^33^ to a target figure of $200 per person-year, the target antiretroviral therapy price for low-resource settings^36^).

### Cost-effectiveness analysis

We calculated the incremental cost-effectiveness ratio (ICER) of home HTC compared with the status quo for both HIV infections and DALYs averted for all simulated criteria for antiretroviral therapy initiation. Sensitivity analyses were done around the discount rate for costs and DALYs averted. All simulations and calculations were done in MATLAB version 2011b.

### Role of the funding source

The funder had no role in study design, data collection, data analysis, data interpretation, or writing of the report. The corresponding author had full access to all the data in the study and had final responsibility for the decision to submit for publication.

## Results

**Figure 2:**
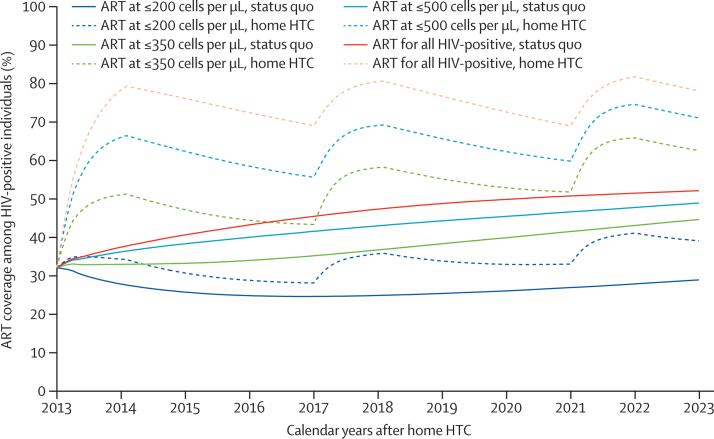
Modelled coverage of antiretroviral therapy Antiretroviral therapy coverage among all HIV-positive individuals over the 10 years after home HTC with changes in antiretroviral therapy eligibility criteria. ART=antiretroviral therapy. HTC=HIV counselling and testing.

Antiretroviral therapy coverage varied with modelled eligibility criteria (figure 2). In the status quo scenario with antiretroviral therapy eligibility of 200 CD4 cells per μL or lower, coverage fell from 32% to 29% (90% model variability 27–30) of HIV-infected individuals over 10 years. Changing of antiretroviral therapy guidelines to 350 cells per μL or lower, 500 cells per μL or lower, and universal antiretroviral therapy increased the coverage to 45% (90% model variability 43–46), 49% (47–50), and 52% (50–54) in the status quo scenarios. Home HTC increased coverage to 39% (90% model variability 37–41), 63% (61–64), 71% (70–72), and 78% (77–79) with increasing antiretroviral therapy eligibility.

**Figure 3:**
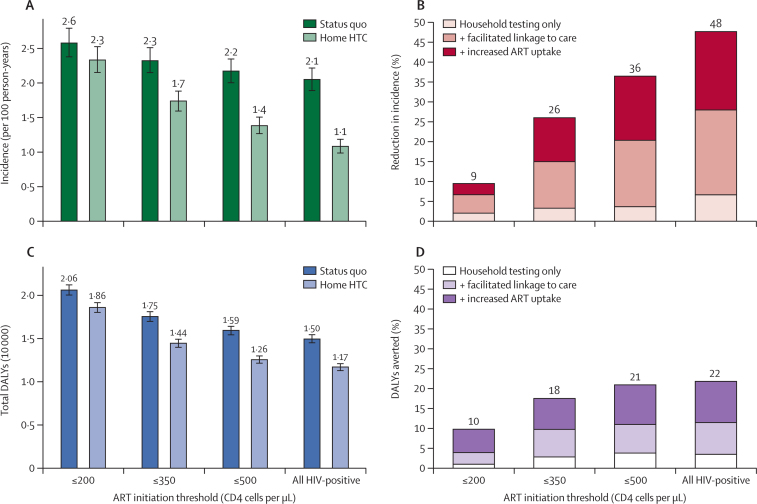
Health effects of home HTC with increasing eligibility criteria for antiretroviral therapy Median incidence and DALYs accrued over 10 years in the model population of mean initial size of 10 000 individuals. In panels B and D, household testing represents immediate HIV testing for all home HTC recipients but no explicit increase in clinic attendance or antiretroviral therapy initiation rates; facilitated linkage to care represents the increase in clinic attendance for HIV-positive individuals following home HTC; and antiretroviral therapy uptake represents the rate of initiation treatment for HIV-positive individuals who are eligible under the modelled guidelines. Error bars represent 90% model variability. (A) Total population incidence averaged over 10 years (mean of 5000 simulations). (B) Decrease in incidence between the baseline scenario (facility-based testing) and the intervention scenario (facility-based testing plus home HTC intervention); figure shows percentage reduction (mean of 5000 simulations) and contribution of different elements of the intervention in the model (mean of 500 simulations). (C) Total DALYs accrued over 10 years (mean of 5000 simulations). (D) DALYs averted between the baseline scenario (facility-based testing) and the intervention scenario (facility-based testing plus home HTC intervention); figure shows percentage reduction (mean of 5000 simulations) and contribution of different elements of the intervention in the model (mean of 500 simulations). HTC=HIV counselling and testing. DALY=disability-adjusted life-year. ART=antiretroviral therapy.

With no intervention, increased antiretroviral therapy threshold alone reduced incidence by 10–21% as eligibility increased (figure 3). Home HTC reduced HIV incidence by 9–48% depending on the antiretroviral therapy threshold (figure 3). The large decreases in incidence at high eligibility thresholds for antiretroviral therapy are possible because many individuals are tested at higher CD4 cell counts, allowing high linkage to care and viral suppression. Total reduction in incidence was mainly driven by facilitated linkage to care (5–21% with increasing eligibility for antiretroviral therapy; figure 3).

Increased eligibility for antiretroviral therapy also reduced DALYs; moving the threshold to 350 cells per μL averted 15%, 500 cells per μL averted 23%, and universal antiretroviral therapy averted 27% compared with the threshold of 200 cells per μL (figure 3). Home HTC reduced DALYs by 10–22% with increasing eligibility for antiretroviral therapy (figure 3). The main contribution to DALYs averted was increased uptake of antiretroviral therapy (6–10% with increasing antiretroviral therapy eligibility; figure 3).

**Figure 4:**
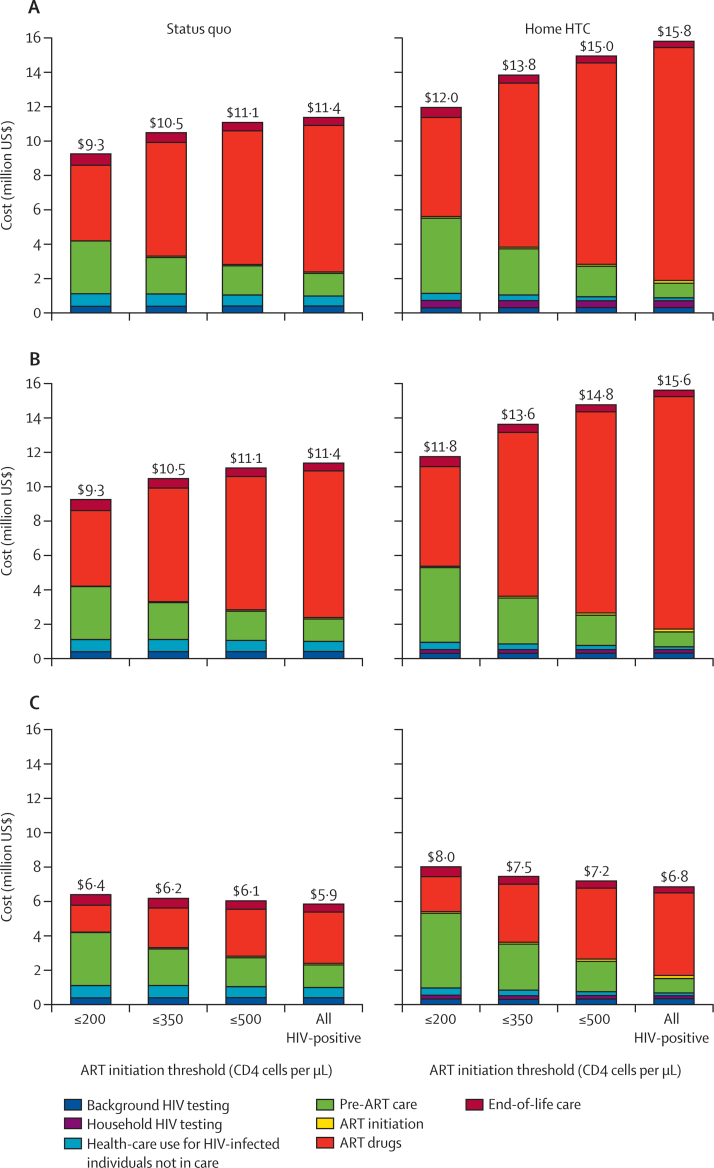
Breakdown of total programme costs Total programme costs accrued over 10 years for the baseline and home HTC model scenarios for a population of mean initial size of 10 000 individuals. (A) Research costing model. (B) Operational costing model with high ART costs. (C) Operational model with low ART costs. HTC=HIV counselling and testing. ART=antiretroviral therapy.

Antiretroviral therapy costs were a major driver of the total programme costs, contributing 24–87% of total costs depending on eligibility criteria (figure 4). The second-largest contributor to total costs was pretreatment costs, including clinical and laboratory testing, and prophylaxis and treatment for opportunistic infections (5–55% with decreasing antiretroviral therapy eligibility). HTC, antiretroviral therapy initiation, end-of-life care, and other health-care costs for HIV-infected individuals not linked to HIV care contributed little to overall costs (<12% each).

The undiscounted additional cost of implementation of home HTC compared with the status quo, for 10 000 adults over 5 years, ranged from $1·57 million (90% model variability $1·44 million to $1·69 million) for an antiretroviral therapy eligibility threshold of 200 cells per μL or lower to $2·93 million ($2·77 million to $3·11 million) for universal antiretroviral therapy (assuming the operational cost model with current antiretroviral therapy prices). Implementation of home HTC was more costly than facility-based testing alone over the 10 year analysis, and did not become cost-saving within this timeframe.

**Figure 5:**
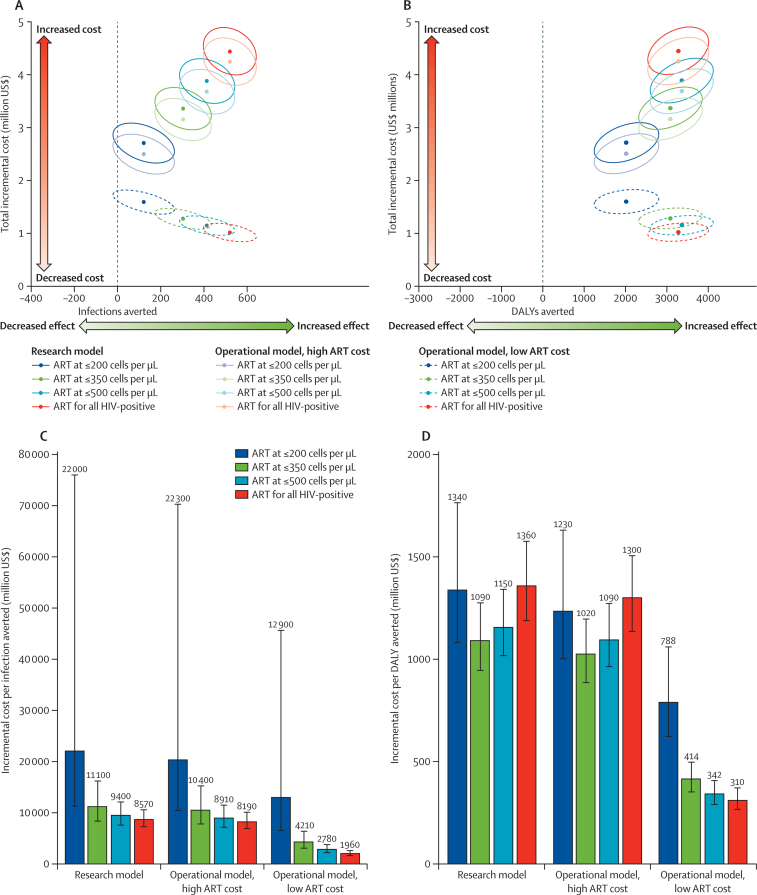
Incremental cost-effectiveness ratios Figure shows ICERs comparing home HTC with status quo at each threshold for antiretroviral therapy initiation. Panels A and B show median of 5000 simulations, with median cost plotted against median effect. Ellipses contain 95% of 5000 simulations; error bars show 90% of the variability in model outputs (5th to 95th percentiles). (A) Infections averted. (B) DALYs averted. (C) Infections averted, by cost model. (D) DALYs averted, by cost model. ICER=incremental cost-effectiveness ratio. HTC=HIV counselling and testing. ART=antiretroviral therapy.

The baseline scenario, with antiretroviral therapy eligibility at 200 cells per μL or lower and the research costing model (figure 5), produced median ICER values of $22 000 per HIV infection averted (90% model variability $11 300–75 900) and $1340 per DALY averted ($1080–1760). A small proportion (2·0%) of the ICER distribution for the incremental cost per infection averted with antiretroviral therapy eligibility at 200 cells per μL or lower were located in the northwest quadrant of the cost-effectiveness plane (figure 5). The incremental cost per infection averted seemed to decrease with expanding eligibility for antiretroviral therapy, but we did not note any clear trend for the incremental cost per DALY averted (figure 5).

The research and operational models with high antiretroviral therapy costs resulted in similar ICER values at all antiretroviral therapy eligibilities. By contrast, decreased cost of antiretroviral therapy reduced ICERs substantially. For example, the ICER per DALY averted was reduced from $1300 (90% model variability $1140–1510) with high costs to $310 ($265–369) at a lower cost, assuming universal antiretroviral therapy (figure 5). Furthermore, with low antiretroviral therapy cost, the trend in ICER values with expansion of eligibility criteria for antiretroviral therapy decreased such that antiretroviral therapy for all HIV-positive individuals became the most cost-effective strategy, although model variability was high.

Sensitivity analyses showed that application of a 3% discount rate to DALYs or varying the cost discount rate from 0% to 12% did not change the qualitative results (appendix).

## Discussion

Expansion of antiretroviral therapy uptake through home HTC is cost effective, with ICERs driven mainly by the cost of drugs. When antiretroviral therapy prices decrease, more expensive testing and linkage strategies, including home HTC, are more likely to be economically viable (panel). Increases in HIV testing and antiretroviral therapy uptake reported from field studies of home HTC have the potential to translate into substantial reductions in HIV morbidity and mortality, and are cost effective at all eligibility criteria for antiretroviral therapy. Our analysis also shows that raising the CD4 count threshold for initiation of antiretroviral therapy from 350 cells per μL to 500 cells per μL could reduce HIV incidence by 6·6% and avert 9·1% of DALYs compared with current practice. However, the potential effect of changes in guidelines might not be fully realised because of low rates of HIV testing for individuals with high CD4 cell counts. By bringing HTC services into the community, home HTC increases testing and linkage to care, thus helping to maximise the number of people benefiting from changes to guidelines. Our findings suggest that raising antiretroviral therapy eligibility from 350 cells per μL to 500 cells per μL with home HTC would reduce incidence by 20% and avert 13% of DALYs. The effect of home HTC seems greater on infections than on DALYs averted over the 10 year timeframe because the probability of HIV transmission from all infected individuals on antiretroviral therapy was reduced, irrespective of their CD4 counts. By contrast, antiretroviral therapy initiation produced a small reduction in morbidity in the model for individuals with CD4 counts of 350 cells per μL and above, because the disability weights were the same and reductions in mortality did not have a substantial effect within the 10 year timeframe of this analysis.^33^

With current prices for antiretroviral therapy in South Africa, total programme costs are driven by the lifetime costs of antiretroviral therapy. Therefore, neither the removal of research-related costs nor task shifting substantially affected cost-effectiveness in our analyses. Even with use of research costs, the most expensive costing model, ICERs per DALY averted are 14–19% (and upper bounds <25%) of the $7350 gross domestic product per person for South Africa in 2012, and can therefore be classed as very cost effective.^44^ ICERs are more favourable when antiretroviral therapy costs are decreased to be similar to global standards,^36^ which could reduce the cost per DALY averted in this programme by 36–76%. This scenario would make home HTC a financially attractive option, particularly at high antiretroviral therapy eligibility criteria.

Our results are robust to changes in task-shifting assumptions, antiretroviral therapy costs, and discounting of rates for costs and health benefits. They are similar to those from a previous cost-effectiveness analysis^27^ done in South Africa, showing that community HTC implemented through mobile vans was very cost effective.

A major strength of this analysis is the pairing of primary data for home testing in South Africa with a detailed model of HIV transmission parameterised from the same study. The model includes detailed data about demographics, risk behaviour, CD4 distribution, antiretroviral therapy initiation, time in pre-antiretroviral therapy care, and antiretroviral therapy coverage. Additionally, findings from detailed microcosting studies allowed us to accurately estimate programme efficiency. However, lack of data for antiretroviral therapy uptake and dropout at increasing eligibility criteria for antiretroviral therapy required us to make assumptions about some treatment-seeking behaviours. We do not include parameter uncertainty in this analysis; model variability represents stochastic variation only. With the small model population size, stochastic variation resulted in large model variability in outputs with small absolute numbers, particularly for infections averted at low thresholds for antiretroviral therapy initiation. This effect would be reduced with a larger population size.

Although the absolute costs of HIV care differ by location, our overall results from this health economic modelling analysis are generalisable to other African settings where community-based interventions have the potential to increase uptake of HTC and linkage to antiretroviral therapy. Findings from a 2010–11 multicountry review^45^ of facility-level costs for antiretroviral therapy showed that South Africa had the highest cost ($682 per patient-year) compared with the average across Ethiopia, Malawi, Rwanda, and Zambia ($208 per patient-year). In high-HIV-prevalence countries with lower antiretroviral therapy costs than in South Africa, community-based HTC and linkage would probably also be cost effective.

This study has important programmatic implications; reductions in lifetime antiretroviral therapy costs can reduce costs per DALY averted by up to 76% and should be a focus for South Africa. By comparison, task shifting from professional counsellors to community health workers saves a relatively small cost, but might be necessary if professional counsellors are not available. Expanding the threshold for antiretroviral therapy initiation, even under the status quo of facility-based HTC, can achieve large reductions in HIV burden, which can be further increased with the addition of home HTC. Insights from this analysis can inform policy makers in South Africa and other African regions with similar HIV burden.

## Figures and Tables

**Table 1:** Unit costs for the household testing intervention

	**Research model**		**Operational model**	
	HIV negative	HIV positive	HIV negative	HIV positive
Personnel	$14·86	$20·10	$5·58	$9·72
Transportation	$1·83	$2·47	$1·14	$2·61
Equipment	$0·18	$0·25	$0·11	$0·19
Supplies	$1·76	$9·94	$0·93	$9·14
Buildings and overhead	$0·89	$1·20	$0·52	$0·91
Start-up	$0·36	$0·49	$0·10	$0·18
Recurring meetings	$0·23	$0·32	$0·12	$0·21
Data capture and use	$0·48	$0·65	$0·19	$0·42
Total (annualised)	$20·59	$35·42	$8·70	$23·37

Table shows per-person costs of HIV testing and linkage through home HTC in 2012 US$. The operational model removes research costs and outputs, incorporates increased testing efficiency from time-and-motion studies, and assumes task shifting from professional counsellors to community care workers.

**Table 2:** Unit costs for all other health-care costs in South Africa

		**Unit cost (2012 US$)**
HIV testing and linkage to care		$20 per diagnostic test
Pre-antiretroviral therapy care		
	CD4 count ≤200 cells per μL	$359 per person-year
	CD4 count 201–350 cells per μL	$238 per person-year
	CD4 count >350 cells per μL	$205 per person-year
Initiation of antiretroviral therapy		
	Patients in pre-antiretroviral therapy care	$95 per initiation*
	Patients not in pre-antiretroviral therapy care	$126 per initiation
	Antiretroviral therapy provision	$565 per person-year
Health-care use for HIV-positive people not linked to care		
	CD4 count ≤200 cells per μL	$167 per person-year
	CD4 count 201–350 cells per μL	$46 per person-year
	CD4 count >350 cells per μL	$13 per person-year
	End-of-life care	$160 per death
Supply-chain management and programmatic support		
	Supply-chain management	20% mark-up on all antiretroviral therapy costs
	Programmatic support†	50% mark-up on all non-antiretroviral therapy costs

Table based on data from Eaton and colleagues.^33^

* In the scenario of universal antiretroviral therapy, pretreatment costs represent health-care costs incurred before initiation of antiretroviral therapy, including assessment of liver, kidney, and bone marrow function.

† The mark-up for programmatic support applies to non-intervention costs only.

## Panel Research in context

**Systematic review**

We searched PubMed and Embase for health economic analyses of community-based HIV counselling and testing (HTC) and linkage to care published between Jan 1, 2000, and June 12, 2014, with the terms “HIV” AND “Africa South of the Sahara” AND (“mass screening” OR “testing” OR “screen” OR “diagnosis” OR “counseling”) AND “cost”. Our results included a 2013 systematic review^10^ which emphasised the heterogeneity between cost-effectiveness studies; these studies are difficult to compare because they include different intervention components delivered via different approaches and costs vary by country and HIV prevalence. We identified 127 abstracts, of which eight studies met our inclusion criteria of community HTC costing studies conducted in sub-Saharan Africa that included testing supplies, personnel, and start-up costs in their estimates.^26–28,37–41^ Costs ranged from US$29·30–31·30 for mobile van HTC in South Africa to $5·00–8·29 for home HTC in Uganda.^37,41^ Church-based HTC in South Africa was found to range from $81·72 to $126·48 (including testing supplies, personnel, utilities, training, buildings, office equipment, and publicity).^25,26^ Averaging across all community HTC strategies, we estimate a cost of $25·00 per person tested (2012 US$) in sub-Saharan Africa. Mathematical modelling has increasingly been used to support analyses of effects and cost-effectiveness of changing treatment policy (recently reviewed by Ying and colleagues^42^), but to our knowledge this analysis is the most detailed pairing of mathematical modelling with cost data for home-based testing done so far. We anticipate with interest the cost-effectiveness analyses that will accompany the large community-randomised controlled trials that are underway,^21–24^ some of which are already using mathematical models to inform and monitor progress.^43^

**Interpretation**

Our findings suggest that home-based HIV testing and counselling for HIV, together with community mobilisation and sensitisation, point-of-care CD4 testing, screening for clinical indicators for antiretroviral therapy initiation, and follow-up visits by a community health worker (to support antiretroviral therapy uptake and adherence) can substantially decrease HIV infections and morbidity when implemented at high coverage. Our cost-effectiveness analyses add to the growing body of evidence that intensive community-based testing strategies will require additional resources but can also be cost effective.
