# Supplementary material for: Cost-effectiveness of community-based strategies to strengthen the continuum of HIV care in rural South Africa: a health economic modelling analysis
Source: Lancet HIV. 2015 Apr;2(4):e159–68. doi: 10.1016/S2352-3018(15)00016-8 (PMC4384819; doi:10.1016/S2352-3018(15)00016-8)
Supplement: Supplementary appendix [file mmc1.pdf]

THE LANCET HIV

Supplementary appendix

This appendix formed part of the original submission and has been peer reviewed. We post it as supplied by the authors.

Supplement to: Smith JA, Sharma M, Levin C, et al. Cost-effectiveness of community-based strategies to strengthen the continuum of HIV care in rural South Africa: a health economic modelling analysis. *Lancet HIV* 2015; published online Feb 24. http://dx.doi.org/10.1016/S2352-3018(15)00016-8.

Online supporting information

1. Model definition

1.1 Overview

We developed an individual-based microsimulation model of adults (ages 18 and over) in a community in rural KwaZulu-Natal (KZN). The aim of the analysis was to evaluate the health impact and cost-effectiveness of community-based HIV testing and counseling (HTC) with enhanced linkage to care through home visits (home HTC) compared to a 'status quo' scenario with ongoing current levels of facility-based HIV testing and ART uptake (SQ; Box 1). The analysis was repeated with expanding ART eligibility criteria of ≤200, ≤350 and ≤500 CD4 cells/mm^3. The model is run with a one-month time step from a standing start at year 2013 for ten years. Home HTC is repeated at years 0, 4 and 8. The model is coded in MATLAB (v2011b, The MathWorks, Natick, Massachusetts) and run for ten years with one-month timesteps.

Box S1. Analysis summary.

Scenario one (baseline, SQ): Background HIV treatment cascade only, encompassing existing facility-based testing. No facilitated referral.

Scenario two (home HTC): Community-based household testing with facilitated referral implemented every four years plus background treatment cascade.

Both scenarios repeated for ART eligibility at ≤200, ≤350, ≤500 and all HIV-positive.

1.2 Population and behaviour

The home HTC pilot study enumerated all individuals in a community of 285 households but only adults aged 18 and over who resided in the household for at least two nights per week were eligible for the intervention. The model simulates all adults in the community irrespective of eligibility (mean community size = 769 adults). Individuals are grouped into households (n=285) with cohabiting and non-cohabiting couples explicitly defined. Household size is randomly allocated according to the distribution observed in the pilot study (Figure S1).

Figure S1. Observed household sizes in the home HTC pilot study.

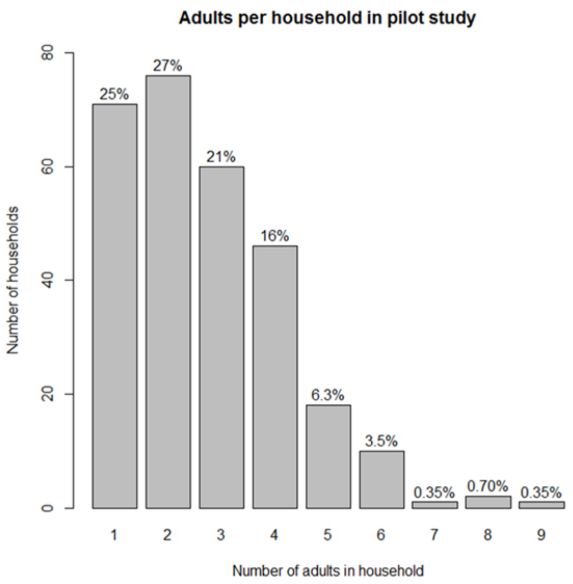

The model replicates the gender and age distribution of the community recorded in the pilot survey (Table S1) stratified according to position in the household: household head, cohabiting partner of household head or other members of the household. First, each household is assigned a household head, 37% of whom are assumed to be male.[1] Each household head is randomly assigned an age category from the observed distribution and their specific age is generated from a uniform distribution across the whole category.

Table S1. Distribution of age and gender in the pilot study community.

	Age group (years)								
	18-19	20-44	25-34	35-44	45-54	55-64	65-74	75-84	85+
All eligible adults									
Men, *n*	22	43	53	34	28	23	12	6	1
Proportion	0.10	0.19	0.24	0.15	0.13	0.10	0.05	0.03	0.00
Women, *n*	42	69	124	66	58	58	16	11	5
Proportion	0.09	0.15	0.27	0.14	0.13	0.13	0.03	0.02	0.03
Household heads only									
Men, *n*	0	4	14	17	21	19	10	6	1
Proportion	0.00	0.04	0.15	0.18	0.23	0.21	0.11	0.07	0.01
Women, *n*	0	4	12	20	26	41	7	11	11
Proportion	0.00	0.03	0.09	0.15	0.20	0.31	0.05	0.08	0.08

Table S2. Age difference between household heads and their cohabiting partners or other household members.

Age difference (years)									
Household head is younger		Same age	Household head is older						
(-11)-(-49)	(-10)-(-1)	0	1-10	11-20	21-30	31-40	41-50	51-60	61-80
Cohabiting partners (proportion)									
0.066	0.11	0.082	0.57	0.066	0.082	0.016	0	0	0
Other household members (proportion)									
0.027	0.058	0.0068	0.11	0.14	0.27	0.22	0.086	0.041	0.041

The main partner of the household head was a cohabitant in 26.3% of all households; these are all assumed to be female partners of male household heads. The remaining individuals in each household

are modeled as other household members, 33.2% of whom are male. The ages of cohabiting partners and other household members are randomly assigned from a discrete distribution relative to the age of the household head (Table S2).

Study eligibility

In the pilot study, n=90 out of 769 individuals (21% of men and 8% of women) enumerated in the household survey were ineligible or did not consent to the intervention. These are disproportionately represented by men and heads of households, and in the model we assume that 80% of the non-intervention men are household heads who are doing migrant work. Migrant workers are considered to be present in the community for one month in every twelve where they form partnerships and contribute to transmission within the community. For the remainder of the time they do not engage in any sex acts with any long-term partner within the study community, but both they and any long-term partner are subject to an elevated risk of short-term partnership formation. Short-term partners who are external to the community are not explicitly modeled but are assumed to share the same age- and gender-specific HIV prevalence as that observed within the community in the baseline survey (Table S3).

Table S3. Age-specific HIV prevalence and incidence.

Age (years)	Prevalence (%)	
	Men	Women
18-19	0.00	16.67
20-24	4.65	29.85
25-34	26.83	52.83
35-44	30.43	72.22
45-54	21.43	47.37
55-64	57.14	25.00
65-74	0.00	0.00
75-84	25.00	0.00
≥85	0.00	7.14

Partnerships, coital frequency and condom use

The model simulates heterosexual transmission only, and sexual partnerships can be formed between any two adults of the opposite sex with a preference for the male partner to be slightly older than the female (modal age difference = 0-4 years in study data, unpublished).

Stable, or long-term, partnerships have a mean duration of 7.15 years in the data and we assume these can only be formed within the modelled community (either within the same household or with an adult in a different household). New long-term partnerships are formed dynamically by matching the number of stable partnerships within the community to the data (Figure S2). Short-term partnerships have a mean duration of three months and are preferentially formed with other adults within the community. Short-term partnership formation is demand-driven by either men or women (usually men who report higher numbers of partners, Table S4). If no adults are available within the community, short-term partners outside the community may be sought. These external adults are not explicitly modelled but have a probability of HIV infection based on the age and gender distribution of HIV prevalence within the study community (Table S3).

Individuals may have a maximum of two concurrent partners at any time, only one of which may be long-term. The monthly probability of an existing partnership dissolving is calculated from the mean partnership duration using a negative exponential distribution. We assume that there is a minimum two-month lag period between cessation of a partnership and formation of the next of the same type.

Figure S2. Distribution of partner numbers at model initiation.

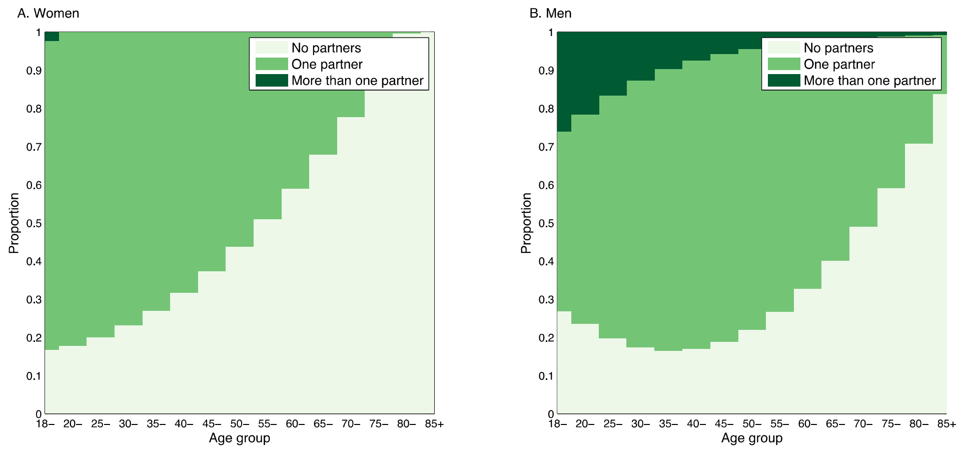

Table S4. Age- and sex-specific distributions of the number of current partners reported in the pilot study. We fit quadratic equations of the form $y = ax^2 + bx + c$ to the proportion of men and women reporting zero partners (where x is age and y is the proportion of that age group with no partners), weighted by the frequency of each age group. For men, a=2.79x10^{-4}, b=-2.14x10^{-2} and c=0.576. For women, a=1.67x10^{-4}, b=-3.86x10^{-3} and c=0.181. Point estimates were taken at the mid-point of each age category. We fit exponential equations of the form $y = ae^{bx}$ to the proportion of men and women reporting more than one partner (where x is age and y is the proportion of that age group with multiple partners), weighted by the frequency of each age group. For men, a=0.714 and b=0.053. For women, a=2.71x10^{13} and b=-1.83. Point estimates were taken at the mid-point of each age category. The point estimates for zero and >1 partners for each age group were then subtracted from one to estimate the proportion of individuals with one partner (Figure S2). These distributions are used for all women except those who are in a within-household relationship, who unanimously report one long-term partner only. Men in a within-household relationship are constrained to have at least one partner.

	Age group (years)														
	15-	20-	25-	30-	35-	40-	45-	50-	55-	60-	65-	70-	75-	80-	≥85
Proportion with no partners															
M	0.45	0.19	0.13	0.09	0.13	0.16	0.29	0.50	0.13	0.29	0.33	0.67	0.50	1.00	0.00
F	0.24	0.25	0.11	0.19	0.17	0.40	0.41	0.26	0.55	0.89	0.60	0.83	1.00	1.00	0.93
Proportion with more than one partner															
M	0.27	0.21	0.16	0.09	0.13	0.16	0.00	0.00	0.06	0.00	0.00	0.00	0.00	0.00	0.00
F	0.02	0.00	0.00	0.00	0.00	0.03	0.00	0.00	0.00	0.00	0.00	0.00	0.00	0.00	0.00

NOTE. M: male, F: female.

Coital frequency with stable partners can vary month-to-month with a range based on the distribution of data from the South African sites of the Partners in Prevention HSV/HIV Transmission Study (http://depts.washington.edu/uwicrc/research/studies/pip_transmission.html). Individuals are randomly allocated into quartiles at the start of the simulation and the number of sex acts each month is sampled randomly from within that category under a uniform distribution (Table S5). All individuals are evaluated in a random order that is re-randomised every time-step, and the coital frequency used that month is the number assigned to whichever partner is evaluated first. We assume all short-term partnerships involve three sex acts per month, also based on the Partners HSV/HIV data and following the assumption made by Hallett *et al.*[2]

Table S5. Coital frequency for long-term partnership (from serodiscordant couples in HSV2/HIV SA data). We assume that one-quarter of women and men aged more than 65 years are sexually active with a maximum of two sex acts per month. All numbers are rounded to the nearest integer.

Age	Men				Women			
	Q1	Q2	Q3	Q4	Q1	Q2	Q3	Q4
<25	1-3	3-6	6-8	8-14	0-3	3-5	5-10	10-28
25-34	0-3	3-7	7-12	12-20	0-3	3-5	5-8	8-16
35-44	0-3	3-6	6-12	12-15	0-2	2-4	4-8	8-29
45-54	0-1	1-2	2-3	3-8	0-2	2-3	3-4	4-9
55-64	1-2	2-3	3-4	4-5	1-2	2-3	3	3
65+	0	0	0	0-2	0	0	0	0-2

Table S6. Proportion of individuals who used a condom at last sex by HIV and relationship status.

	In a long-term relationship (married or living with partner)	Not in a long-term relationship (single, divorced or widowed)
Uninfected	0.100	0.304
HIV-positive	0.343	0.423

We assume a 'take' pattern of condom use, where individuals either use condoms consistently or not at all. Condom use at last sex was significantly associated with HIV infection status at baseline (uninfected: 26.0%, HIV-positive: 41.7%, p<0.001) and relationship status (married or living with partner: 15.0%, single, widowed or divorced: 36.7%, p<0.001). We replicate this joint distribution at model initialization (Table S6) and individuals are reassigned when they test HIV-positive, form or dissolve a long-term partnership. In the study data, condom use at last sex was also significantly associated with age (p<0.001) but we do not capture this in the model. Condom use was not associated with gender (p=0.31).

Population turnover

Children and teenagers enumerated in the pilot survey enter the model as they reach age 18 years at a mean rate of 0.0025 per adult per month using Poisson random generation. Background (non-HIV) mortality rates for men and women are the result of fitting an exponential function ($y = \alpha e^{\beta x}$) to the estimates in an original analysis on South African survival data by Dorrington *et al.* (http://www.mrc.ac.za/bod/1999report.pdf), as performed by Hallett *et al.*[2,3] Parameters are (α=0.0015, β=0.062) for men and (α =0.0004, β =0.080) for women (where *y* is the mortality rate and *x* is the exact age). Survival probabilities for each member of any partnership are modeled independently.

1.3 HIV infection & transmission

Biological parameters

The baseline transmission probability per sex act, β_0, is set at 0.1%[4], and different HIV-related cofactors are applied according to each individual's attributes and behavior. Condom use and male circumcision reduce HIV transmission by 78% and 65% per sex act, respectively.[4,5] Coinfections representing HSV2 and other STIs are assumed to increase HIV acquisition by a factor of 3.4 for women and 2.8 for men[6] and transmission by a factor of two (HSV2 is associated with an 0.18 increase in log VL[7] which equates to approximately 50% increase in infectiousness using [4]). STIs are initially distributed by age and gender according to the observed HSV2 prevalence in sub-Saharan Africa and all individuals are subject to an STI incidence rate, which is evaluated every six months (Table S7).

Table S7. Age-specific STI prevalence and incidence.[8,9]

Age (years)	Prevalence (%)		Incidence (per 100py)	
	Men	Women	Men	Women
15-19	7.3	16.1	1.96	3.91
20-24	13.5	27.2	1.73	2.69
25-29	18.8	34.1	1.51	1.84
30-34	23.1	38.9	1.32	1.27
35-39	27.6	43.1	1.20	0.89
40-44	31.9	46.7	1.09	0.64
≥45	36.2	48.5	0.99	0.45

HIV natural history

On infection, individuals progress to one of four CD4 cell categories with the probabilities given in Table S8. The CD4 count then progresses through each subsequent category until death. The mean years spent in each CD4 cell count category were derived from Lodi et al 2011[10] and a pooled-analysis of

African observational cohort studies[11] by Cori *et al.* (2014).[12] For individuals with CD4 cell counts below 200, the mean years in the category can be interpreted as mean survival time. The infectiousness rate ratio is given relative to an individual with CD4 cell count above 350[13] (in Donnell *et al.*, there is no substantial difference between transmission of those with CD4 cell counts 350-500 or 500+ so these categories were combined here).

Table S8. Progression, relative infectivity and duration of CD4 categories.

		Probability of immediate progression on infection*	Infectiousness rate ratio	Mean years in category	Monthly probability of progression
CD4 cell count if not treated					
	500+	0.58	1	6.37	0.0130
	350-500	0.23	1	2.86	0.0287
	200-350	0.16	1.59	3.54	0.0233
	≤200	0.03	4.99	2.30	0.0356
On ART	N/A	N/A	0.08	Life expectancy same as uninfected	Age-related, as for uninfected

*Fitted to data in Lodi *et al.* (2011) by Cori *et al.* (2014).[10,12]

Male circumcision

Fifteen per-cent of all men are assumed to be circumcised; male circumcision status was not significantly associated with age in the pilot study (p=0.88) so this was applied uniformly across all ages and is assumed to be constant through time.

Model fitting

Age- and gender-specific incidence in the model was manually fitted to the pattern of incidence observed in rural KwaZulu-Natal in an independent population survey (Table S9).[14,15] For each gender

and age group, the baseline transmission probability per sex act β_0 was multiplied by a fitting cofactor, F. This cofactor is intended to represent all age- and gender-related differences in incidence as well as accounting for any misreporting of behavioural factors in the study data.

Table S9. Age-specific HIV incidence in KZN and fitting cofactor.[14,15]

Age (years)	Incidence (per 100 py)		Fitting cofactor, F	
	Men	Women	Men	Women
18-19	0.74	4.43	0.05	4.00
20-24	2.53	6.49	0.10	4.00
25-29	4.43	5.51	0.40	12.5
30-34	2.91	3.52	0.65	4.26
35-39	3.16	2.26	0.38	2.00
40-44	1.71	2.23	0.30	1.15
45-49	0.95	0.66	0.25	1.35
50-55	0.90	0.40	0.18	0.36
≥55	0.90	0.40	0.20	0.20

1.4 Background: HIV testing, linkage to care and ART initiation

Background care cascade overview

All individuals may progress through a background care cascade comprising facility-based HIV testing, linkage to care and ART initiation regardless of whether or not they have been exposed to the home HTC intervention (Figure S3). Progression rates between these stages are given in the form of a monthly probability of progression from one state to another. These are based on the recorded coverage of HIV testing, pre-ART clinic visit and ART use at the pilot and phase II baseline surveys and are detailed in Tables S8 and S10-S12.

Figure S3. Treatment cascade in status quo scenario.

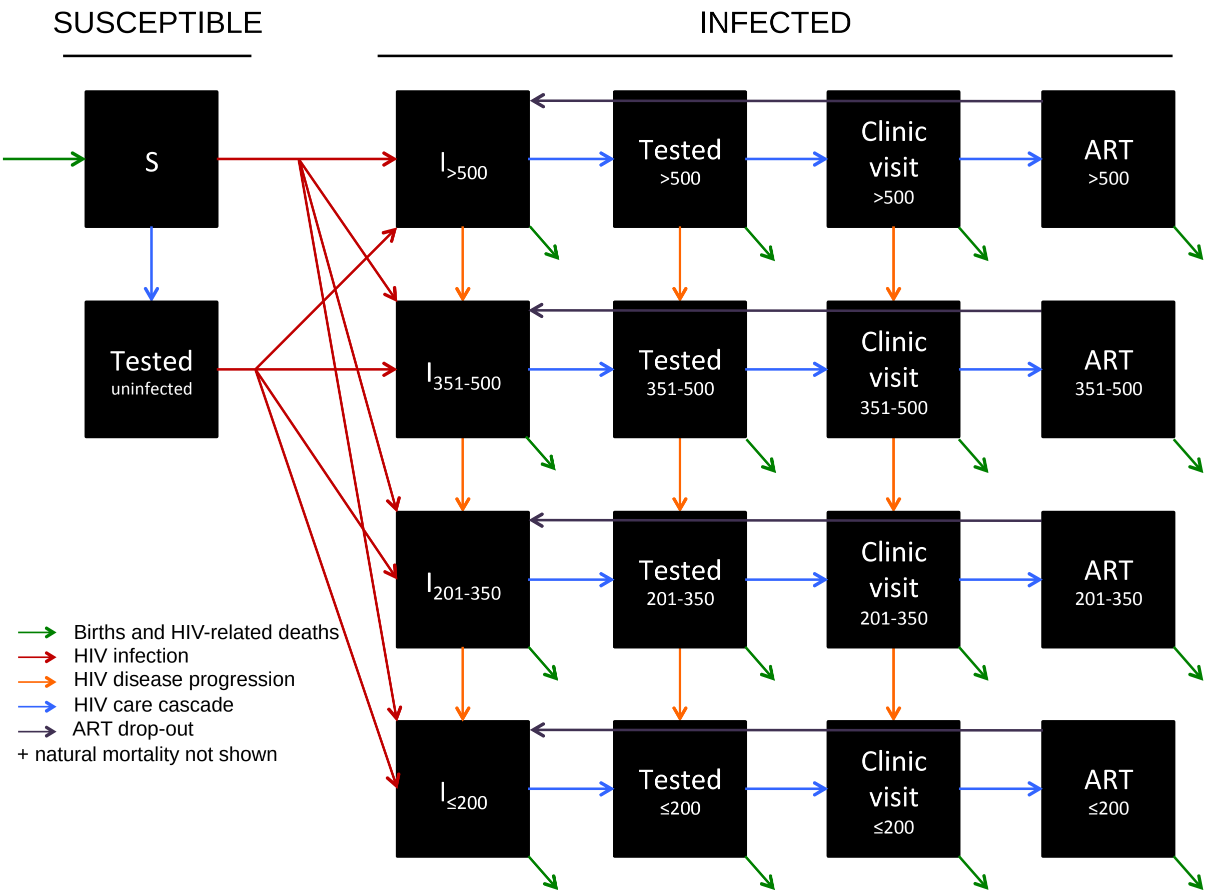

HIV testing

The background coverage of HIV testing is specific to sex, age and infection status and is derived from the phase II baseline data (Table S10). This is used in the 'status quo' scenario, as well as during non-intervention years and for individuals who have not received the intervention in the home HTC scenario. The observed coverage of having ever tested for HIV at baseline is multiplied by the proportion of those tested whose test was in the preceding year (41.0% for uninfected individuals and 69.7% for individuals who are HIV-positive but not on ART, irrespective of sex or age group) to estimate the proportion of individuals who test in one year. The monthly probability of HIV testing is then estimated using equation (eq.) 1. This method assumes that background HIV testing rates do not change over time and that all infected individuals will eventually get tested, unless they die first.

$$ProbHIVTest_{i,j,k} = HIVTestInLastYear_k * EverTestedForHIV_{i,j,k} * \left(1 - e^{-\frac{1}{12}}\right) \quad \text{(eq. 1)}$$

Table S10. HIV testing parameters by age, sex and HIV status.

Age group (*j*)	Women (*i*=1)			Men (*i*=2)		
	Uninfected (*k*=1)	CD4 >200 (*k*=2)	CD4 ≤200 (*k*=3)	Uninfected (*k*=1)	CD4 >200 (*k*=2)	CD4 ≤200 (*k*=3)
	Study data: HIV testing coverage (% ever tested)					
18-19	47.2	60.0	100.0	31.0	40.0	100.0
20-24	75.3	76.9	100.0	35.9	53.9	100.0
25-29	81.9	77.4	92.6	46.1	50.0	80.0
30-34	79.3	67.5	97.6	46.6	42.1	88.2
35-39	78.8	61.8	97.7	39.3	57.1	75.0
40-44	70.6	61.5	96.7	54.1	45.5	90.0
45+	48.3	40.0	95.1	45.4	46.2	85.7
	Model: per-month probability of HIV test					
18-19	0.0155	0.0334	0.0557	0.0102	0.0223	0.0557
20-24	0.0247	0.0429	0.0557	0.0118	0.0300	0.0557
25-29	0.0269	0.0431	0.0516	0.0151	0.0279	0.0446
30-34	0.0260	0.0376	0.0544	0.0153	0.0235	0.0492
35-39	0.0258	0.0344	0.0539	0.0129	0.0318	0.0418
40-44	0.0232	0.0343	0.0539	0.0177	0.0253	0.0502
45+	0.0158	0.0223	0.0530	0.0149	0.0257	0.0478

Linkage to care: pre-ART clinic visit

The baseline coverage for linkage to care, which we define as a pre-ART clinic visit, is specific to CD4 category status only and is derived from the pilot baseline data. We estimate a monthly probability from the observed proportions of individuals who have linked to care (all of whom have had an HIV test; eq. 2, Table S11). This method assumes that the rate of attending the clinic does not vary over time and that these individuals have attended the clinic in the past year. All infected individuals who have had an HIV test will eventually visit the clinic, unless they die first. We assume no change in the probability of linkage to care with expanding ART eligibility criteria.

$$PrLinkage_k = Linkage_k \left(1 - e^{-\frac{1}{12}}\right) \qquad \text{(eq. 2)}$$

Table S11. CD4-specific parameters for linkage to care.

	CD4 category			
	>500	351-500	201-350	≤200
Study data: HIV-positive individuals ever visited clinic	22.9%	44.2%	53.1%	86.3%
Model: per-month probability of clinic visit for HIV-positive individual	0.018	0.035	0.043	0.069

ART initiation

We assume that all individuals on ART at the time of the pilot baseline survey initiated therapy with a CD4 count of ≤200 cells/mm^3. Therefore background ART initiation in the baseline scenario is only possible when an individual's CD4 count falls below this threshold. When the threshold is elevated to ≤350 cells/mm^3, ≤500 cells/mm^3 and universal access for the alternative analyses, per-month ART initiation probabilities are assumed to halve for eligible individuals with CD4 351-500 cells/mm^3 and halve again for >500 cells/mm^3. We also assume that individuals must visit the clinic before ART

initiation can take place. All infected and eligible individuals who have had a clinic visit will eventually initiate ART, unless they die first.

After initiation, ART takes three months to reach full efficacy and infectiousness decays exponentially over this period to reach an overall reduction in infectivity of 96%.[16] Mortality on ART varies by the CD4 cell count at initiation and by the time since initiation (Table S12).[17-19]

Table S12. CD4-specific parameters for ART coverage and initiation.

	Current CD4 category			
	>500	351-500	201-350	≤200
Study data: HIV-positive individuals on ART at baseline*	0%	0%	0%	81.3%
Model: per-month probability of ART initiation given HIV testing and linkage	0.0162†	0.0325†	0.0650†	0.0650
Model: mortality rate in first year post-ART initiation (per year)	0.013	0.025	0.050	0.100
Model: mortality rate after first year post-ART initiation (per year)	0.013	0.013	0.025	0.050

*assumed that all individuals currently on ART initiated treatment with CD4 ≤200 cells/mm^3

†when eligible under assumed ART initiation guidelines

Drop-out from an ART program is fixed at 10% per year in the first year of treatment and 5% per year thereafter for individuals who initiate ART through the background care cascade or 5% per year throughout for individuals who initiate ART following home HTC. Following ART drop-out, we assume that an individual's CD4 count reverts to its category prior to initiation. HIV-infected individuals cannot re-link to care until their CD4 count has fallen below 200 cells/mm^3, unless they receive the home HTC intervention, irrespective of the ART initiation threshold.

1.5 Intervention: community-based home HTC with facilitated referral

The home HTC intervention acts to strengthen the HIV care cascade via an immediate HIV test for all eligible and consenting adults together with increased linkage to care and ART uptake. It is implemented three times over the ten-year model run in the form of three rounds of simultaneous expanded HIV testing at 0, 4 and 8 years. All eligible and consenting adults receive an immediate HIV test unless they are already on ART, or have had an HIV test within the previous six months (we assume that the latter group are likely to withhold consent to the intervention). In the pilot study, HIV-positive individuals then received a point-of-care CD4 test and were referred to a local HIV clinic. In the model, all home HTC-related changes last for one year post-intervention, after which testing and treatment-seeking behaviour returns to that observed in the SQ arm.

In the pilot study, n=90 out of 769 individuals (11.7%) were ineligible or did not consent to HIV testing, represented disproportionately by men and heads of households. We assumed that 80% of the non-tested men are household heads who are doing migrant work to derive a joint distribution of intervention recipients that we use in the model (Table S13).

Table S13. Combined probability of eligibility, consent and knowledge of HIV test result.

	Household head	Non-household head
Women	0.805	0.991
Men	0.667	0.922

Linkage to care following home HTC

Following the home HTC intervention, 68% of HIV-positive individuals who were not previously linked to care had visited a clinic within a month, 89% within three months and 95% by the end of six months. Clinic attendance following home HTC was not associated with CD4 count (*p*=0.087), age (*p*=0.84) or

gender (p=0.80). To estimate the monthly probability of clinic attendance we fit an exponential distribution of the form $y = A(1 - e^{-\mathrm{rt}})$ to this data using non-linear least squares (eq. 3).

$$pLinkage_{HomeHTC} = 0.932\left(1 - e^{-\frac{15.3}{12}}\right) \quad \text{(eq. 3)}$$

Here, $pLinkage_{HomeHTC}$ is per-month probability of an HIV-positive individual visiting clinic for the first time following the home HTC visit. This gives an estimated monthly probability of 67% linkage to care following home HTC and we assume this does not vary in analyses with expanded ART initiation criteria.

ART initiation following home HTC

CD4 counts at ART initiation were not recorded in the pilot study and CD4 measurements taken during intervention follow-up visits measure reconstituted CD4 cell levels for individuals on ART. We assumed that 90% of individuals who started ART following home HTC had a CD4 count of ≤200 cells/mm^3 at initiation (other indicators for ART initiation include TB coinfection and pregnancy, which are not included in the model).

We fitted an exponential distribution to the proportion of HIV-positive individuals initiating ART to estimate the monthly probability of ART initiation post-home HTC (eq. 4). Time to ART initiation was calculated from the time of each individual's clinic visit assuming that this occurred at the mid-point between consecutive follow-up visits.

$$pART_{HomeHTC} = 1 - e^{-\frac{-4.22}{12}} \quad \text{(eq. 4)}$$

Here, $pART_{HomeHTC}$ is the per-month probability of an eligible, consenting and HIV-positive individual initiating ART following linkage to care after the home HTC visit. When the ART initiation threshold is elevated to ≤350 cells/mm^3, ≤500 cells/mm^3 and universal access for the alternative analyses, we use the same assumption as for non-intervention treatment initiation that per-month ART initiation probabilities are assumed to halve for eligible individuals with CD4 351-500 cells/mm^3 and halve again for >500 cells/mm^3 (Table S14).

Table S14. ART coverage and initiation post-intervention.

	CD4 category			
	>500	351-500	201-350	≤200
Model: per-month probability of ART initiation	0.074†	0.148†	0.296†	0.296

†when eligible

We do not assume any changes in condom use or other sexual behavior relating to the intervention but condom use is associated with knowledge of HIV status (Table S6), therefore increased testing will lead to increased condom use in the population overall. Sensitivity analyses indicate that this does not significantly contribute to the overall intervention impact (data not shown).

1.6 Quantifying health states

Disability-adjusted life years (DALYs) are attached to each HIV-related health state and these are summed over all individuals for the duration of the model runtime. We use the same disability weights as described in Eaton, Menzies *et al.* (Table S15). [20]

Table S15. Disability weights for health states in the model.

Status	Disability weight
Uninfected	0
HIV infected: CD4 cell count 500+	0.053
HIV infected: CD4 cell count 350-500	0.053
HIV infected: CD4 cell count 200-350	0.221
HIV infected: CD4 cell count ≤200	0.547
On ART: First year, CD4 at initiation >200	0.053
On ART: First year, CD4 at initiation ≤200	0.053
On ART: Subsequent years	0.053
Deceased	1

DALYs: AIDS cases, receiving ARV treatment, from Salomon *et al.* (2012).[21] No category in GBD classification for uninfected with CD4 >350, assumed the same as HIV-positive on ART.[20]

1.7 Cost estimates

Costs were collected onsite during November 2013 in KwaZulu Natal, South Africa from the Linkages study, a community randomized trial of community HIV counseling and testing. Time and motion observation of home HTC was conducted to determine staff time and resource utilization per home visit and also to facilitate removal of research time and costs for the operational cost estimate. We observed than HTC takes an average of 17 minutes per HIV- and 50 minutes per HIV+ person tested respectively. After accounting for travel time, follow-ups, paperwork, and other staff responsibilities, we estimated that a community care worker could test 7 HIV- persons per day or 5 HIV+ persons per day (with the average number tested changing depending on HIV prevalence). We assumed a program of 20 community care workers and 4 supervisory nurses. Staff were assumed to work 7 hours per day, 215 days per year after accounting for national holidays, sick days, and paid vacations. Supervisory staff were expected to test 2-3 persons per day. In the research model, we used the number tested per day of 4 HIV- persons and 3 HIV+ persons observed in the pilot study. Total program costs were divided by the number of persons tested by HIV status under each scenario to determine the cost per person tested. Supply costs per person tested included gloves, HIV screening test kit, lancet, cotton balls, alcohol swabs, and results card. Additional supplies for HIV+ persons tested included confirmatory test, tie breaker test (assumed to be used in 5% of all HIV+ cases), and point of care CD4 test.

Table S16. HTC testing volume assumptions: Operational model.

Employee type	# staff	# days worked/ year	# tested/ day if all persons are HIV-	# tested/ day if all persons are HIV+	Annual # tested if all are HIV+ (lower bound)	Annual # tested if all are HIV- (upper bound)
Community care workers	20	215	7	5	21500	30100
Senior nurses	4	215	3	2	1720	2580
Total	24		10	7	23220	32680

Table S16. HTC testing volume assumptions: Research model.

Employee type	# staff	# days worked/ year	# tested/ day if all persons are HIV-	# tested/ day if all persons are HIV+	Annual # tested if all are HIV+ (lower bound)	Annual # tested if all are HIV- (upper bound)
Community care workers	20	215	4	3	12900	17200
Senior nurses	4	215	3	2	1720	2580
Total	24		7	5	14620	19780

2. Model validation

Figure 2A compares the median HIV incidence over ten years in the model under the former ART eligibility criteria of ≤200 CD4 cell mm^{-3} to an independent survey from KwaZulu-Natal under the same national guidelines.[14] The predicted age-specific pattern in the model closely matches the observed data.

Figure S4. Model incidence

Age-specific incidence over ten years with ART initiation at ≤200 CD4 cells mm^{-3}. Solid lines and error bars show the incidence with 95% confidence interval (CI) reported in Kwa-Zulu Natal in Tanser *et al* (2013). Dashed lines and block colours show the median and 90% variability in model outputs.

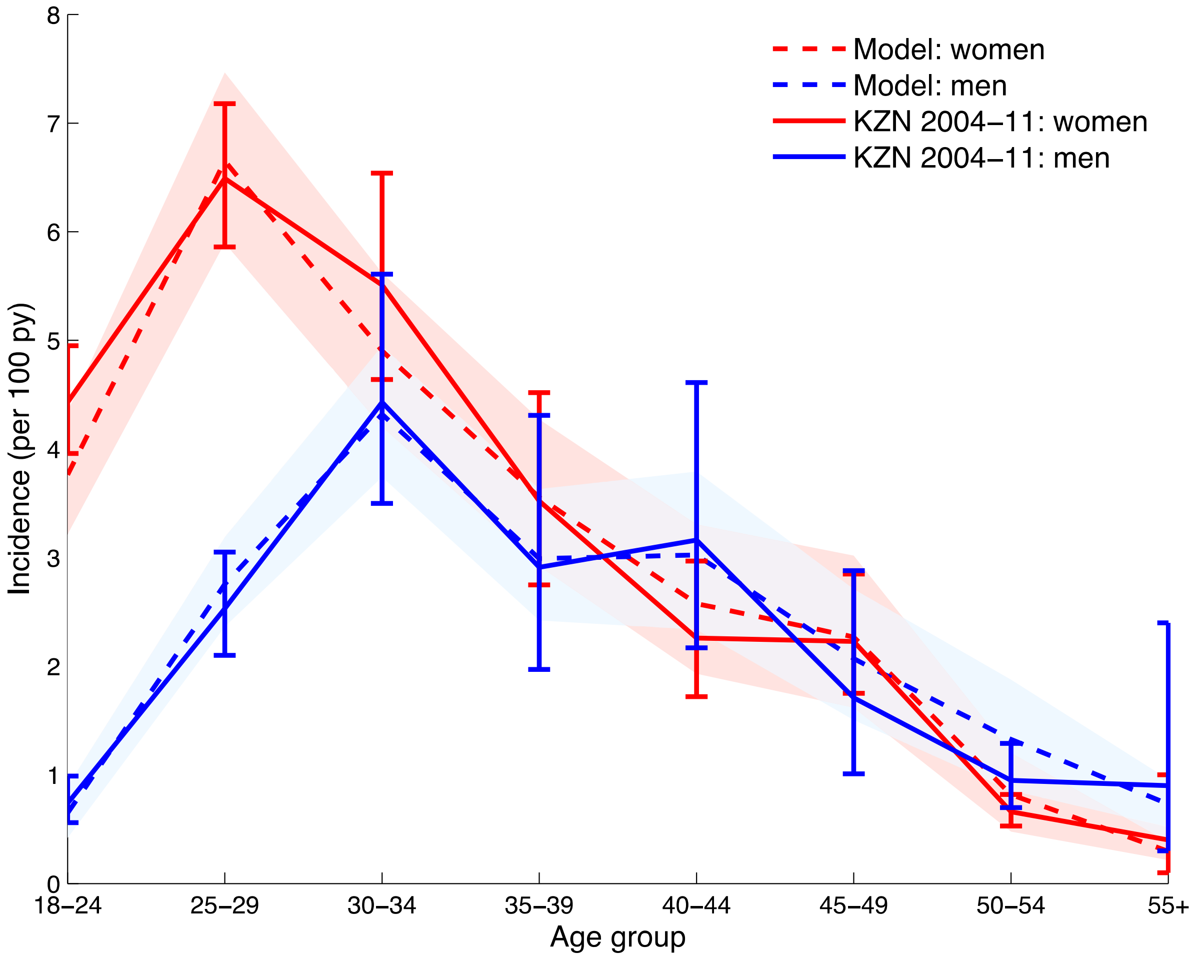

Figure S5. Model validation.

A. Age and gender distribution at model initiation.
B. Model incidence over ten years.
C. CD4 category distribution over ten years with no intervention and ART eligibility at CD4 ≤200 cells/mm^3.
D. CD4 category at ART initiation.
E. Source of HIV transmissions over ten years by CD4 category with ART eligibility at CD4 ≤200 cells/mm^3.
F. Source of HIV transmissions over ten years by partnership type with ART eligibility at CD4 ≤200 cells/mm^3.

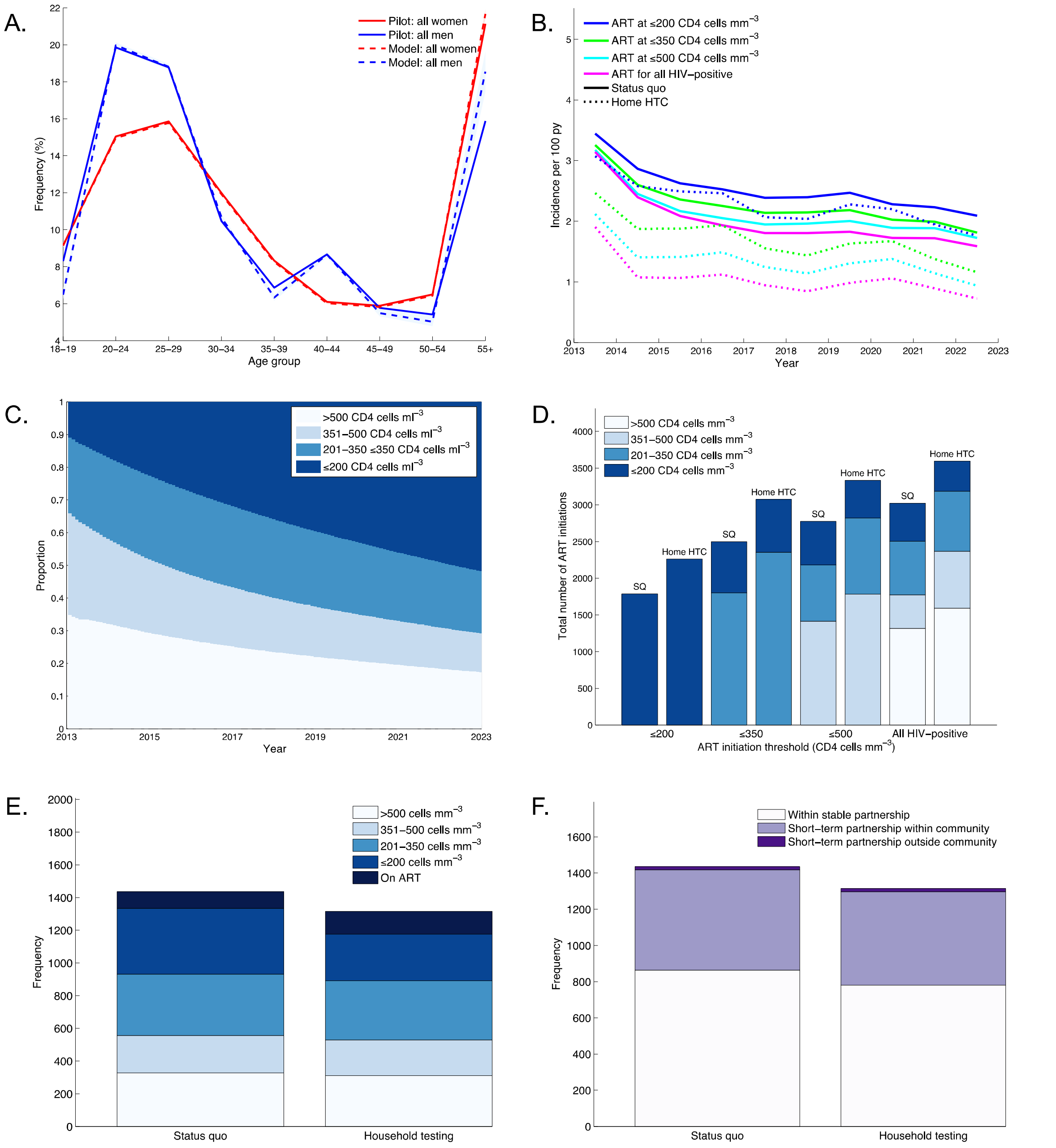

3. Sensitivity analyses

DALY discount rate

DALYs are not discounted in the base-case scenario; we compare this to a 3% annual discount rate for all DALYs, implemented in the base case model. Table S17 shows that discounting DALYs at 3% per year increases the incremental cost per DALY averted by 18%-20% and increasing the cost discount rate from 3% to 6% decreases ICERs by 12-14%.

Table S17. Effect of DALY and cost discount rates on incremental cost per DALY averted. Costs calculated using the baseline cost model (research costs).

DALY discount rate	ART eligibility criteria (cells/mm^3)	Cost discount rate			
		Non-discounted	3%	6%	12%
Non-discounted	≤200	1548 (1253-2042)	1337 (1082-1765)	1164 (942-1536)	902 (730-1189)
	≤350	1261 (1094-1475)	1089 (945-1275)	948 (823-1109)	734 (637-859)
	≤500	1336 (1176-1552)	1155 (1016-1341)	1005 (884-1167)	778 (685-904)
	All	1571 (1376-1823)	1358 (1189-1575)	1182 (1035-1371)	915 (801-1062)
3%	≤200	1849 (1497-2434)	1597 (1293-2103)	1390 (1126-1830)	1077 (872-1417)
	≤350	1502 (1305-1758)	1298 (1127-1519)	1130 (981-1322)	875 (760-1024)
	≤500	1593 (1402-1851)	1376 (1211-1599)	1198 (1054-1392)	928 (816-1078)
	All	1870 (1641-2170)	1616 (1418-1875)	1407 (1234-1632)	1089 (956-1264)

References

1. van Rooyen H, Barnabas RV, Baeten JM, et al. High HIV Testing Uptake and Linkage to Care in a Novel Program of Home-Based HIV Counseling and Testing With Facilitated Referral in KwaZulu-Natal, South Africa. *JAIDS Journal of Acquired Immune Deficiency Syndromes* 2013; **64**(1): e1-e8 10.1097/QAI.0b013e31829b567d.
2. Hallett TB, Baeten JM, Heffron R, et al. Optimal Uses of Antiretrovirals for Prevention in HIV-1 Serodiscordant Heterosexual Couples in South Africa: A Modelling Study. *PLoS Medicine* 2011; **8**(11): e1001123.
3. Dorrington R, Bradshaw D, Wegner T. Estimates of the Level and Shape of Mortality Rates in South Africa Around 1985 and 1990 Derived by Applying Indirect Demographic Techniques to Reported Deaths. 1999. http://www.mrc.ac.za/bod/1999report.pdf (accessed 20th May 2013).
4. Hughes JP, Baeten JM, Lingappa JR, et al. Determinants of Per-Coital-Act HIV-1 Infectivity Among African HIV-1–Serodiscordant Couples. *Journal of Infectious Diseases* 2012; **205**(3): 358-65.
5. Weiss HA, Halperin D, Bailey RC, Hayes RJ, Schmid G, Hankins CA. Male circumcision for HIV prevention: from evidence to action? *AIDS* 2008; **22**(5): 567-74.
6. Glynn J, Biraro S, Weiss H. Herpes simplex virus type 2: a key role in HIV incidence. *AIDS* 2009; **23**(12): 1595-8.
7. Barnabas RV, Webb EL, Weiss H, Wasserheit JN. The role of coinfections in HIV epidemic trajectory and positive prevention: a systematic review and meta-analysis. *AIDS* 2011; **25**(13): 1559-73.
8. Looker KJ, Garnett GP, Schmid GP. An estimate of the global prevalence and incidence of herpes simplex virus type 2 infection. *Bulletin of the World Health Organisation* 2008; **86**(10): 805-12.
9. Programme C. Global & Regional Population Pyramids. http://collen.ageing.ox.ac.uk/data/international/global-population-trends/ (accessed August 1 2014).
10. Lodi S, Phillips A, Touloumi G, et al. Time From Human Immunodeficiency Virus Seroconversion to Reaching CD4+ Cell Count Thresholds <200, <350, and <500 Cells/mm3: Assessment of Need Following Changes in Treatment Guidelines. *Clinical Infectious Diseases* 2011; **53**(8): 817-25.
11. Duration from seroconversion to eligibility for antiretroviral therapy and from ART eligibility to death in adult HIV-infected patients from low and middle-income countries: collaborative analysis of prospective studies. *Sexually Transmitted Infections* 2008; **84**(Suppl 1): i31-i6.
12. Cori A, Ayles H, Beyers N, et al. HPTN 071 (PopART): A Cluster-Randomized Trial of the Population Impact of an HIV Combination Prevention Intervention Including Universal Testing and Treatment: Mathematical Model. *PLoS ONE* 2014; **9**(1): e84511.
13. Donnell D, Baeten JM, Kiarie J, et al. Heterosexual HIV-1 transmission after initiation of antiretroviral therapy: a prospective cohort analysis. *The Lancet* 2010; **375**(9731): 2092-8.
14. Tanser F, Bärnighausen T, Grapsa E, Zaidi J, Newell M-L. High Coverage of ART Associated with Decline in Risk of HIV Acquisition in Rural KwaZulu-Natal, South Africa. *Science* 2013; **339**(6122): 966-71.
15. Wallrauch C, Barnighausen T, Newell ML. HIV prevalence and incidence in people 50 years and older in rural South Africa. *South African Medical Journal* 2010; **100**(12): 812-4.
16. Cohen MS, Chen YQ, McCauley M, et al. Prevention of HIV-1 Infection with Early Antiretroviral Therapy. *New England Journal of Medicine* 2011; **365**(6): 493-505.
17. Kitahata MM, Gange SJ, Abraham AG, et al. Effect of Early versus Deferred Antiretroviral Therapy for HIV on Survival. *New England Journal of Medicine* 2009; **360**(18): 1815-26.
18. Etard JF, Ndiaye I, Thierry-Mieg M, et al. Mortality and causes of death in adults receiving highly active antiretroviral therapy in Senegal: a 7-year cohort study. *AIDS* 2006; **20**(8): 1181-9.
19. Mahy M, Lewden C, Brinkhof MWG, et al. Derivation of parameters used in Spectrum for eligibility for antiretroviral therapy and survival on antiretroviral therapy. *Sexually Transmitted Infections* 2010; **86**(Suppl 2): ii28-ii34.

20. Eaton JW, Menzies NA, Stover J, et al. Health benefits, costs, and cost-effectiveness of earlier eligibility for adult antiretroviral therapy and expanded treatment coverage: a combined analysis of 12 mathematical models. *The Lancet Global Health* 2014; **2**(1): e23-e34.
21. Salomon JA, Vos T, Hogan DR, et al. Common values in assessing health outcomes from disease and injury: disability weights measurement study for the Global Burden of Disease Study 2010. *The Lancet* 2012; **380**(9859): 2129-43.
